# The novel estrogenic receptor GPR30 alleviates ischemic injury by inhibiting TLR4-mediated microglial inflammation

**DOI:** 10.1186/s12974-018-1246-x

**Published:** 2018-07-12

**Authors:** Zengli Zhang, Pei Qin, Youliang Deng, Zhi Ma, Hang Guo, Haiyun Guo, Yushu Hou, Shiquan Wang, Wangyuan Zou, Yanyuan Sun, Yulong Ma, Wugang Hou

**Affiliations:** 1 Department of Anaesthesiology, Xijing Hospital, The Fourth Military Medical University, Xi’an, 710032 China; 2 Department of Anaesthesiology, Xiangya Hospital, Central South University, Changsha, 410008 China; 3 Anaesthesia and Operation Center, Chinese PLA General Hospital, Beijing, 100853 China; 4 Department of Anaesthesiology, Xi’an Children’s Hospital, Xi’an, 710003 China; 5 Department of Anesthesiology, The First Affiliated Hospital of Xi’an Jiaotong University, Xi’an, 710061 China; 6 Department of Anaesthesiology, PLA Army General Hospital, Beijing, 100700 China

**Keywords:** Stroke, Estrogen, GPR30, Microglial inflammation, TLR4

## Abstract

**Background:**

The steroid hormone estrogen (17-β-estradiol, E2) provides neuroprotection against cerebral ischemic injury by activating estrogen receptors. The novel estrogen receptor G protein-coupled receptor 30 (GPR30) is highly expressed in the brain and provides acute neuroprotection against stroke. However, the underlying mechanisms remain unclear.

**Methods:**

In this study, ovariectomized female mice were subjected to middle cerebral artery occlusion (MCAO), and E2, G1, and ICI182780 were administered immediately upon reperfusion. The infarction volume, neurological scores, and neuronal injuries were examined. Primary microglial cells were subjected to oxygen-glucose deprivation (OGD), and the drugs were administered immediately upon reintroduction. The pro-inflammatory cytokines TNF-α, IL-1β, and IL-6 in penumbra and microglia were assessed by ELISA. The cell viability and lactose dehydrogenase (LDH) release of neurons co-cultured with microglia were analyzed using cell counting kit-8 (CCK8) and LDH release assays. Microglial activation as well as GPR30, Iba1, and Toll-like receptor 4 (TLR4) protein expression and TLR4 mRNA expression were detected. Additionally, NF-κB activity was detected in lipopolysaccharide (LPS)-activated microglia after the activation of GPR30.

**Results:**

GPR30 was highly expressed in microglia and significantly increased after ischemic injury. The activation of GPR30 significantly reduced the infarction volume, improved the neurological deficit, and alleviated neuronal injuries. Moreover, GPR30 activation significantly reduced the release of TNF-α, IL-1β, and IL-6 from ischemic penumbra and microglia subjected to OGD and alleviated neuronal injury as assessed using the CCK8 and LDH assays. Finally, the activation of GPR30 relieved microglial activation, reduced Iba1 and TLR4 protein expression and TLR4 mRNA levels, and inhibited NF-κB activity.

**Conclusions:**

Microglial GPR30 exerts acute neuroprotective effects by inhibiting TLR4-mediated microglial inflammation, which indicates that GPR30 may be a potential target for the treatment of ischemic stroke.

**Electronic supplementary material:**

The online version of this article (10.1186/s12974-018-1246-x) contains supplementary material, which is available to authorized users.

## Background

Estrogen confers strong neuroprotection against cerebral ischemic injury in rodents and humans via estrogenic receptors α and β (ERα, ERβ) and G protein-coupled receptor 30 (GPR30) [1–3]. However, the peripheral actions of estrogen on reproductive organs and the potential to increase the risk of endometrial and breast cancer limit its clinical application [4].

GPR30 is a novel estrogenic receptor that is highly expressed in the brain [5, 6]. Our team members originally found that the GPR30 agonist G1 reduced ischemia reperfusion injury in ovariectomized (OVX) mice [7]. Moreover, an acute bolus dose of G1 following ischemic injury significantly enhanced neuronal survival and decreased cell apoptosis [8, 9]. Thus, GPR30 plays an important role in the acute neuroprotective effect of estrogen. However, the explicit molecular mechanisms underlying the acute neuroprotective effects of GPR30 remain unclear.

Toll-like receptor 4 (TLR4) is primarily expressed in microglia and mediates microglial activation [10]. The inhibition of TLR4 has beneficial effects on tissue homeostasis and attenuates both myocardial and spinal cord ischemic injury [11, 12]. E2 (17-β-estradiol) and G1 reduced the TLR4 levels in peripheral macrophages, resulting in anti-inflammatory effects [13], suggesting that GPR30 participates in the peripheral anti-inflammatory pathway. Therefore, we investigated the roles of microglial GPR30 in the inflammatory pathway of the central nervous system (CNS), the neuroprotective effects of estrogen against ischemic injury, and the potential molecular mechanisms.

## Methods

### Animals

One hundred and seventy 6- to 8-week-old female C57BL/6 mice (20 ± 2 g) were obtained from the Laboratory Animal Center of Fourth Military Medical University. All animal experimental procedures were approved by the Ethics Committee for Animal Experimentation of the Fourth Military Medical University and followed the National Institutes of Health Guide for the Care and Use of Laboratory Animals (https://grants.nih.gov/grants/olaw/Guide-for-the-Care-and-use-of-laboratory-animals.pdf).

### Cell cultures and neuron-microglia co-cultures

Primary mouse microglial cultures were harvested from 1- to 2-day-old neonatal C57BL/6 pups. Briefly, after removal of the meninges and hippocampus, the cortical tissues were subjected to enzymatic digestion and mechanical isolation. Then, the mixed cortical cells were passed through a 70-μm nylon mesh cell strainer and seeded into a cell culture flask in Dulbecco’s Modified Eagle’s Medium (DMEM, HyClone) containing 10% FBS (Gibco) and 1% penicillin/streptomycin. Fourteen to 15 days later, the mixed glial cultures were shaken on an orbital shaker at 220 rpm for 1 h. Then, the detached microglial cells in the supernatant were collected and reseeded into cell culture containers. The purity of the microglia in culture was more than 95%, as confirmed by staining with the microglial marker Iba1 (see Additional file 1: Figure S1).

Neuronal cultures were harvested from the cerebral cortices of E16–E17 mouse embryos. Dissociated cells were obtained and then seeded onto poly-D-lysine-coated culture plates or glass cover slips. The Neurobasal Medium (Gibco) contained 2% B27 (Invitrogen), 1% glutamine, and 1% penicillin and streptomycin. The purity of the neuronal cultures was more than 95%, as confirmed by staining with neuronal and glial markers.

For the indirect neuron-microglia co-cultures, neurons were seeded in 24-well plates and incubated for 3 days. Then, primary microglia (microglia:neurons = 1:2) were added to 0.4-μm pore-sized Transwell inserts (Costar, USA). The neurons and microglia were co-cultured for another 2 days before oxygen-glucose deprivation (OGD) treatment.

### Groups and drug treatment

In vivo, all mice were ovariectomized (OVX) to eliminate the influence of circulatory estrogen. After 1 week, all mice except for those in the sham group were subjected to middle cerebral artery occlusion (MCAO) injury. Then, mice were randomly divided into the following groups: sham, vehicle (cottonseed oil), E2, G1, and ICI182780+E2. The drugs E2 (17-β-estradiol), G1 (GPR30 agonist), and ICI182780 (ERα/β antagonist) were administered immediately upon reperfusion. In particular, 235 μl of E2 (1 mmol/l) was administered via intraperitoneal injection, and 2 μl of G1 (0.1 g/l) and 2.8 μl of ICI182780 (2 mmol/l) were administered via left intracerebroventricular infusion.

In vitro, primary microglial cells were randomly divided into the following groups: control, vehicle (dimethyl sulfoxide, DMSO), E2, G1, and ICI182780+E2. All cells except for those in the control group were subjected to OGD treatment. E2 (10 nmol/l), G1 (10 nmol/l), and ICI182780 (1 μmol/l) were administered immediately upon reintroduction. In addition, primary microglial cells were randomly divided into three groups: control, vehicle (DMSO), and G1. All cells except for those in the control group were exposed to lipopolysaccharide (LPS, 10 ng/ml) for 12 h to induce microglial inflammation. G1 (10 nmol/l) was administered immediately upon the end of the LPS exposure.

The drugs E2 (#10006315, Cayman Chemical, USA), G1 (#10008933, Cayman Chemical, USA), and ICI182780 (#10011269, Cayman Chemical, USA) were dissolved in cottonseed oil and dimethyl sulfoxide for in vivo and in vitro experiments, respectively, and LPS (Sigma) was dissolved in cell culture medium. All drugs were administered as previously described [7, 14–17]. All experiments followed the principles of randomization and double blindness.

### Ovariectomization of the female mice (OVX)

All mice were subjected to OVX to eliminate the influence of circulatory estrogen on the results, and the operation was conducted as previously described [7]. Briefly, mice were anesthetized with 10% chloral hydrate (0.03 ml/kg), and the ovaries were then exposed via a midline dorsal skin and muscle layer incision at the right or left side of the vertebral column. After ligating the uterine horn, the ovaries were cut, and the wounds were closed.

### Intracerebroventricular cannulation and infusion

The lateral ventricle was targeted − 0.2 mm anterior-posterior, + 1.0 mm medial-lateral, and − 2.2 mm dorsal-ventral from the bregma. Cannulas (62003, RWD Life Science) were held in the left hemispheres of the brain [7]. Mice were given 7 days to recover from the surgery. Afterward, the mice were subjected to MCAO injury, and drugs were injected into the lateral ventricle at a rate of 1 μl/min immediately upon reperfusion.

### Middle cerebral artery occlusion (MCAO)

MCAO was conducted as previously described [7]. General anesthesia was induced by 10% chloral hydrate (0.03 ml/kg), and the body temperatures of the OVX female mice were maintained at 37.0–37.5 °C. A midline skin incision was performed to expose the left common carotid artery (CCA). Then, a nylon monofilament was inserted from the left CCA to the origin of the MCA. The regional cerebral blood flow (rCBF) was monitored using laser Doppler flowmetry (Periflux System 5000, PERIMED, Stockholm, Sweden). Mice were included only if blood flow was reduced to more than 20% of the preischemic baseline level during ischemia and was restored to up to 70% of the preoperative level during reperfusion. After 1 h of MCAO, cerebral blood flow was recovered by removing the filament. The physiological parameters are provided in Additional file 1.

### Neurobehavioral evaluation

Neurobehavioral evaluation was performed 24 h after reperfusion according to a previous report [7]. The neurological evaluation was scored using a 5-point scale: 0, no neurological deficit; 1, failure to extend left forepaw fully; 2, circling to the left; 3, inability to bear weight on the left; 4, no spontaneous walking with depressed level of consciousness. The performer was blind to the treatments.

### Assessment of infarct volume

Infarct volume was assessed by 2,3,5-triphenyl-2H-tetrazoliuM chloride (TTC) staining as previously described [7]. Briefly, mice were decapitated, and their brains were rapidly removed and cut into 1-mm-thick coronal brain slices. Then, the slices were immersed in 2% TTC at 37 °C until an obvious color change appeared, transferred to 4% paraformaldehyde for 6 h of fixation, and photographed using a digital camera (Canon IXUS 220HS). The percent infarct was calculated as [(*V*_C_ *− V*_L_)/*V*_C_] × 100, where *V*_C_ is the volume of the control hemisphere and *V*_L_ is the volume of the noninfarcted tissue in the lesioned hemisphere.

### Nissl staining

Nissl staining was applied to observe morphological changes in cells within the ischemic penumbra. The brains were perfused with cold 4% paraformaldehyde. Then, the brains were removed after post-fixation and dehydrated in 20 and 30% sucrose solutions. Subsequently, 10-μm-thick sections were prepared using the Leica CM1900 frozen slicer. The experimental steps were strictly performed according to the manufacturer’s manual of the Nissl staining kit (#G1430, Solarbio, China). The cells with large cell bodies, a rich cytoplasm, and one or two large, round nuclei were intact neurons, while the cells with shrunken cell bodies, condensed nuclei, dark cytoplasm, and empty vesicles were damaged neurons, as described in the manufacturer’s manual. Cells in five different fields were counted.

### Terminal deoxynucleotidyl transferase-mediated dUTP nick-end labeling (TUNEL) staining

TUNEL staining was performed using an In Situ Cell Death Detection Kit (Roche Diagnostics, Mannheim, Germany) according to the manufacturer’s instructions. TUNEL-positive neurons/DAPI were regarded as an apoptosis index.

### OGD/R

Briefly, the cells were seeded in DMEM without glucose (Gibco), transferred into a modular incubator chamber, and flushed with 3 l/min of a 95% N_2_ and 5% CO_2_ gas mixture for 15 min at room temperature. The chamber was then sealed and placed in a 37 °C container. OGD was carried out for 1, 2, or 4 h. Following OGD, the cells were incubated with normal media for an additional 12 h of reperfusion under normal conditions.

### CCK-8 cell viability assay

A Cell Counting Kit (Seven Sea Biotech, Shang Hai, China) was used to assess cell survival. The experimental steps were strictly performed according to the manufacturer’s manual. Briefly, 50 μl of CCK-8 solution was added to 500 μl of medium solution in each neuronal culture well of a 24-well plate and incubated for 4 h at 37 °C. The absorbance at 450 nm was measured with a microplate reader (Infinite M200, TECAN).

### LDH release assay

The LDH-Cytotoxicity Colorimetric Assay Kit II (#K313-500, BioVision, USA) was used to detect cell injury. The experimental steps were strictly performed according to the manufacturer’s manual. Briefly, medium solution (10 μl per well) was added to each neuronal culture well of a 24-well plate in an optically clear 96-well plate. Then, 100 μl of LDH Reaction Mix was added to each well, mixed, and incubated for 30 min at room temperature. The absorbance at 450 nm was measured with a microplate reader (Infinite M200, TECAN).

### Cell fraction assay

Twelve hours after primary microglia were subjected to G1 treatment, the cytoplasmic and nuclear extracts of primary microglial cells were prepared in accordance with the protocol provided in the NE-PER Nuclear and Cytoplasmic Extraction Reagent Kit (Pierce, Rockford, IL, USA). Briefly, 200 μl of ice-cold cytoplasmic extraction reagent I was added to the washed microglia and incubated on ice for 10 min, and 11 μl of ice-cold cytoplasmic extraction reagent II was then added and incubated on ice for 1 min. The tube was then centrifuged for 5 min at maximum speed in a microcentrifuge (16,000×*g*). The supernatant fraction (cytoplasmic extract) was immediately transferred to a clean pre-chilled tube, and 100 μl of ice-cold nuclear extraction reagent was added to the insoluble fraction by vortexing for 15 s every 10 min for a total of 40 min. The tube was centrifuged at maximum speed in a microcentrifuge for 10 min. The nuclear extract fraction was then moved to a clean pre-chilled tube. All extracts were analyzed by Western blotting.

### Quantitative (real-time) polymerase chain reaction (real-time PCR)

Primary microglial cells were collected from each group. Total RNA was isolated using a TaKaRa MiniBEST Universal RNA Extraction Kit (code no. 9767, TaKaRa, Japan) and quantified. Then, 1000 ng of total RNA was reverse transcribed in a 20-μl reaction at 37 °C for 15 min followed by 85 °C for 5 s using PrimeScript RT Master Mix (code no. RR036A, TaKaRa, Japan). Real-time PCR was performed in a 25-μl reaction according to the manufacturer’s manual for SYBR Premix Ex Taq (Tli RNaseH Plus) (#RR820A, TaKaRa, Japan). The TLR4 mRNA primer sequences [TaKaRa Biotechnology (Dalian) Co., Ltd.] were as follows: F: 5′-GGGCCTAAACCCAGTCTGTTTG-3′, R: 5′-CTTCTGCCCGGTAAGGTCCA-3′. The reaction was performed at 95 °C for 3 min followed by 40 cycles of 95 °C for 10 s and 55 °C for 30 s on the IQ5 Multicolor Real-Time PCR Detection System (Bio-Rad, USA). The primers and sequences derived from the relative mRNA expression were analyzed using the formula 2^−(Ct target gene − Ct reference gene)^.

### ELISA

The levels of pro-inflammatory cytokines (TNF-α, IL-1β, IL-6) were determined by ELISAs (R&D Systems, Minneapolis, MN, USA). The experimental steps were strictly performed according to the manufacturer’s manual.

### Immunofluorescence staining assay

Immunofluorescence staining was performed on frozen coronal sections of mouse brains or on primary microglia plated on cover slips. The mouse brains were fixed with 4% paraformaldehyde. After post-fixation and concentration gradient dehydration, the brains were cut into 10-μm-thick sections using a Leica CM1900 frozen slicer. The cells were fixed with 4% paraformaldehyde for 30 min. The brain sections and cell cover slips were washed three times with PBS and then incubated overnight at 4 °C in a humidified atmosphere with primary antibodies. The following primary antibodies were used: mouse anti-TLR4 (1:100; Santa Cruz, USA), rabbit anti-TLR4 (1:100; Santa Cruz, USA), rabbit anti-GPR30 (1:200; Abcam, England), goat anti-Iba1 (1:200; Novus, USA), rabbit anti-Iba1 (1:500; Wako, Japan), and mouse anti-NeuN (1:200; Millipore, USA). Then, the samples were incubated with mixtures of Alexa-488 (red, Invitrogen) or Alexa-594 (red, Santa Cruz) and Alexa-647 (green, Invitrogen)-conjugated donkey anti-goat or anti-rabbit and donkey anti-mouse secondary antibodies for 2 h in the dark at room temperature. Finally, the sections were photographed using an Olympus BX51 (Japan) fluorescence microscope. The average area of single microglial cells was measured by ImageJ software.

### Western blotting

Expression of the GPR30, Iba1, and TLR4 proteins in penumbra and primary microglia was measured by Western blotting as previously described [2]. The extracted proteins were separated by 10% SDS-PAGE and electrically transferred to polyvinylidene difluoride membranes. Then, the membranes were blocked with TBST containing 5% nonfat dry milk for 1 h at room temperature. The following primary antibodies were used: rabbit anti-GPR30 (1:1000; Abcam, England), rabbit anti-Iba1 (1:500; Wako, Japan), rabbit anti-TLR4 (1:200; Santa Cruz, USA), rabbit anti-NF-κB (Cell Signaling Technology; 1:500), mouse anti-β-actin (1:1000; Cell Signaling Technology, USA), rabbit anti-GAPDH (1:1000; Cell Signaling Technology, USA), and rabbit anti-Histone H2A.X (Cell Signaling Technology; 1:1000). The membranes were shaken at 60 rpm/min at 4 °C overnight and incubated with an IRDye secondary anti-rabbit or mouse antibody (Thermo Scientific, USA) for 1 h. Protein bands were visualized using the LI-COR Odyssey System (LI-COR Biotechnology, USA).

### Statistical analysis

GraphPad Prism 7.0 was used for statistical analyses. Data were collected by two independent investigators who were blind to the drug treatments. Comparisons among multiple groups were performed using one-way ANOVA followed by Tukey’s post hoc test. Neurological deficit scores are presented as medians and were analyzed using two-tailed Mann-Whitney *U* tests, and the other values are presented as the mean ± SD. *p* values < 0.05 were considered statistically significant.

## Results

### Activating GPR30 induced neuroprotection against ischemic stroke

**Fig. 1:**
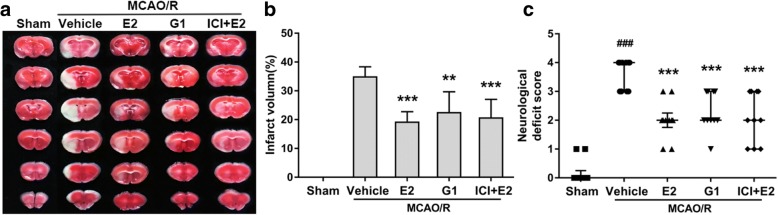
Neuroprotection of GPR30 against ischemic stroke. **a** Representative photographs of brain slices showing the infarct volume assessed 24 h after reperfusion in OVX mice after MCAO/R injury. **b** Infarction volume as a percentage of the sham value. The data are expressed as the mean ± SD and analyzed by one-way ANOVA with Tukey’s post-test. **p < 0.01, ***p < 0.001 compared with the vehicle group. *n* = 6 per group. **c** Neurological deficit scores evaluated 24 h after reperfusion in OVX mice after MCAO/R injury. The data are expressed as the median and were analyzed by the Mann-Whitney *U* test. ^###^*p* < 0.001 compared with the sham group. ***p < 0.001 compared with the vehicle group. *n* = 10 per group. OVX mice ovariectomized mice, MCAO/R middle cerebral artery occlusion/reperfusion

Compared with the sham group, the GPR30 expression levels in the penumbra gradually increased and peaked 24 h after ischemia reperfusion (Additional file 1: Figure S2). The antagonist ICI182780 was used to block the functions of ERα and ERβ. MCAO/R injury resulted in a large infarct volume in OVX female mice (Fig. 1a). Following the administration of E2, G1, and ICI182780 + E2 immediately upon reperfusion, the infarct volume significantly decreased (*p* ˂ 0.0001, vehicle vs E2; *p* = 0.0010, vehicle vs G1; *p* = 0.0002, vehicle vs ICI + E2; Fig. 1a, b), and the neurological deficit was dramatically relieved (*p* = 0.0002, vehicle vs E2; *p* = 0.0008, vehicle vs G1; *p* = 0.0008, vehicle vs ICI + E2; Fig. 1c).

**Fig. 2:**
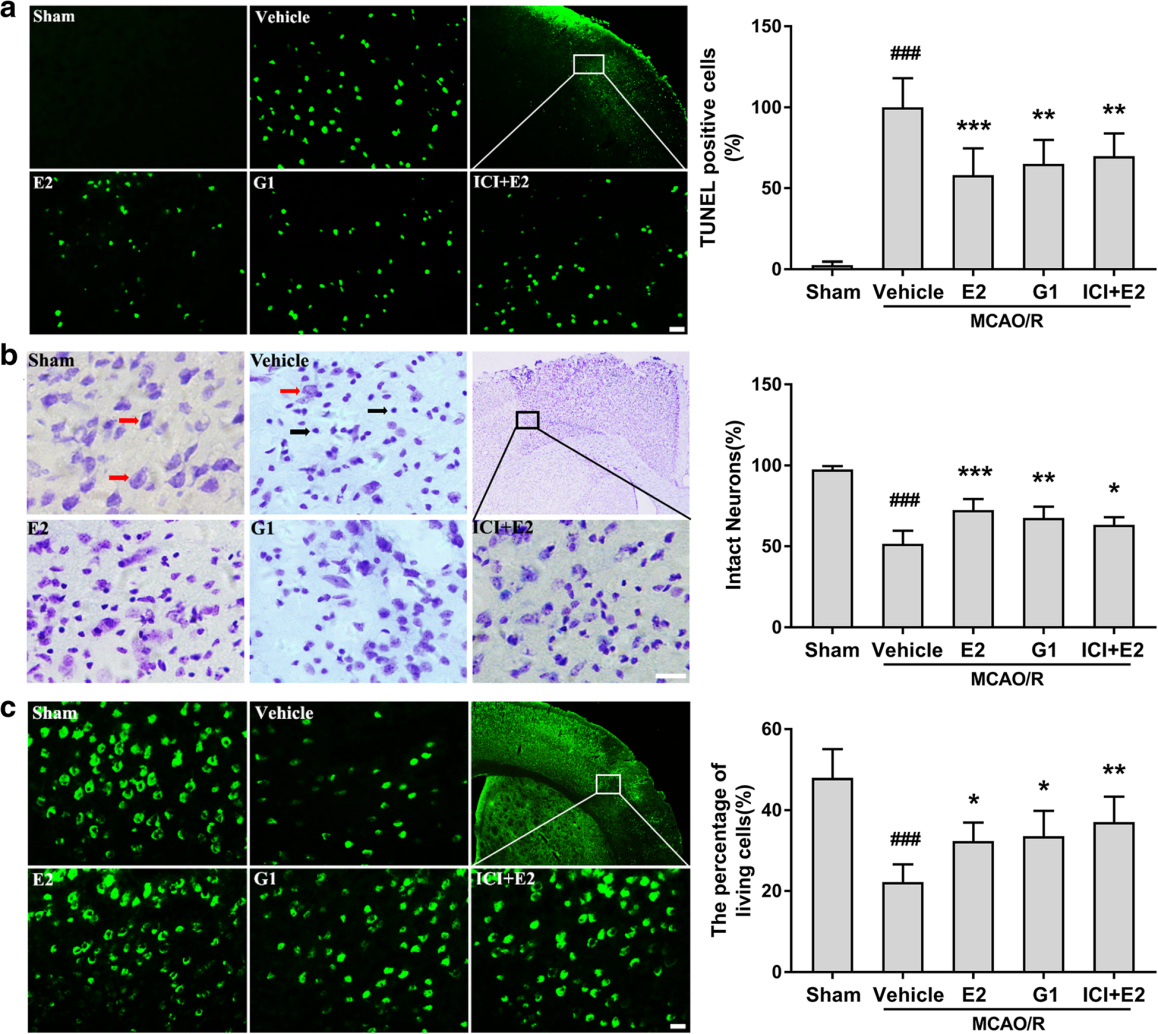
GPR30 activation alleviates neuronal injury in the ischemic penumbra. **a** Left: representative photomicrographs showing TUNEL staining in the ischemic penumbra of OVX mice 24 h after reperfusion. Scale bars = 20 μm. Right: the percentage of TUNEL-positive cells in the ischemic penumbra. The data are expressed as the mean ± SD and analyzed by one-way ANOVA with Tukey’s post-test. ^###^*p* < 0.001 compared with the sham group. **p < 0.01, ***p < 0.001 compared with the vehicle group. *n* = 6 per group. **b** Left: Nissl staining showing morphological neuronal changes in the ischemic penumbra of OVX mice 24 h after reperfusion. The red arrows indicate intact neurons with large cell bodies, rich cytoplasms, and one or two large round nuclei, while the black arrows indicate damaged neurons with shrunken cell bodies, condensed nuclei, dark cytoplasms, and empty vesicles. Scale bars = 50 μm. Right: the percentage of intact neurons in the ischemic penumbra. The data are expressed as the mean ± SD and analyzed by one-way ANOVA with Tukey’s post-test. ^###^*p* < 0.001 compared with the sham group. *p < 0.05, **p < 0.01, ***p < 0.001 compared with the vehicle group. *n* = 6 per group. **c** Left: NeuN staining showing the survival of neurons in the ischemic penumbra of OVX mice 24 h after reperfusion. Scale bars = 20 μm. Right: the percentage of living neurons in the ischemic penumbra. The data are expressed as the mean ± SD and analyzed by one-way ANOVA with Tukey’s post-test. ^###^*p* < 0.001 compared with the sham group. *p < 0.05, **p < 0.01 compared with the vehicle group. *n* = 6 per group. TUNEL terminal deoxynucleotidyl transferase-mediated 2′-deoxyuridine 5′-triphosphate nick-end labeling

In ischemic penumbra, GPR30 activation by E2 and G1 reduced the numbers of TUNEL-positive neurons (apoptosis) (*p* = 0.0003, vehicle vs E2; *p* = 0.0023, vehicle vs G1; *p* = 0.0094, vehicle vs ICI + E2; Fig. 2a) and injured neurons (*p* ˂ 0.0001, vehicle vs E2; *p* = 0.0011, vehicle vs G1; *p* = 0.0221, vehicle vs ICI + E2; Fig. 2b). According to the NeuN staining results, E2, G1, and ICI182780 + E2 significantly increased the number of NeuN-positive cells (surviving neurons) (*p* = 0.0416, vehicle vs E2; *p* = 0.0192, vehicle vs G1; *p* = 0.0015, vehicle vs ICI + E2; Fig. 2c). No significant differences were observed among the E2, G1, and ICI + E2 groups.

### Activating GPR30 reduced inflammatory cytokine release after MCAO/R and OGD/R injuries

**Fig. 3:**
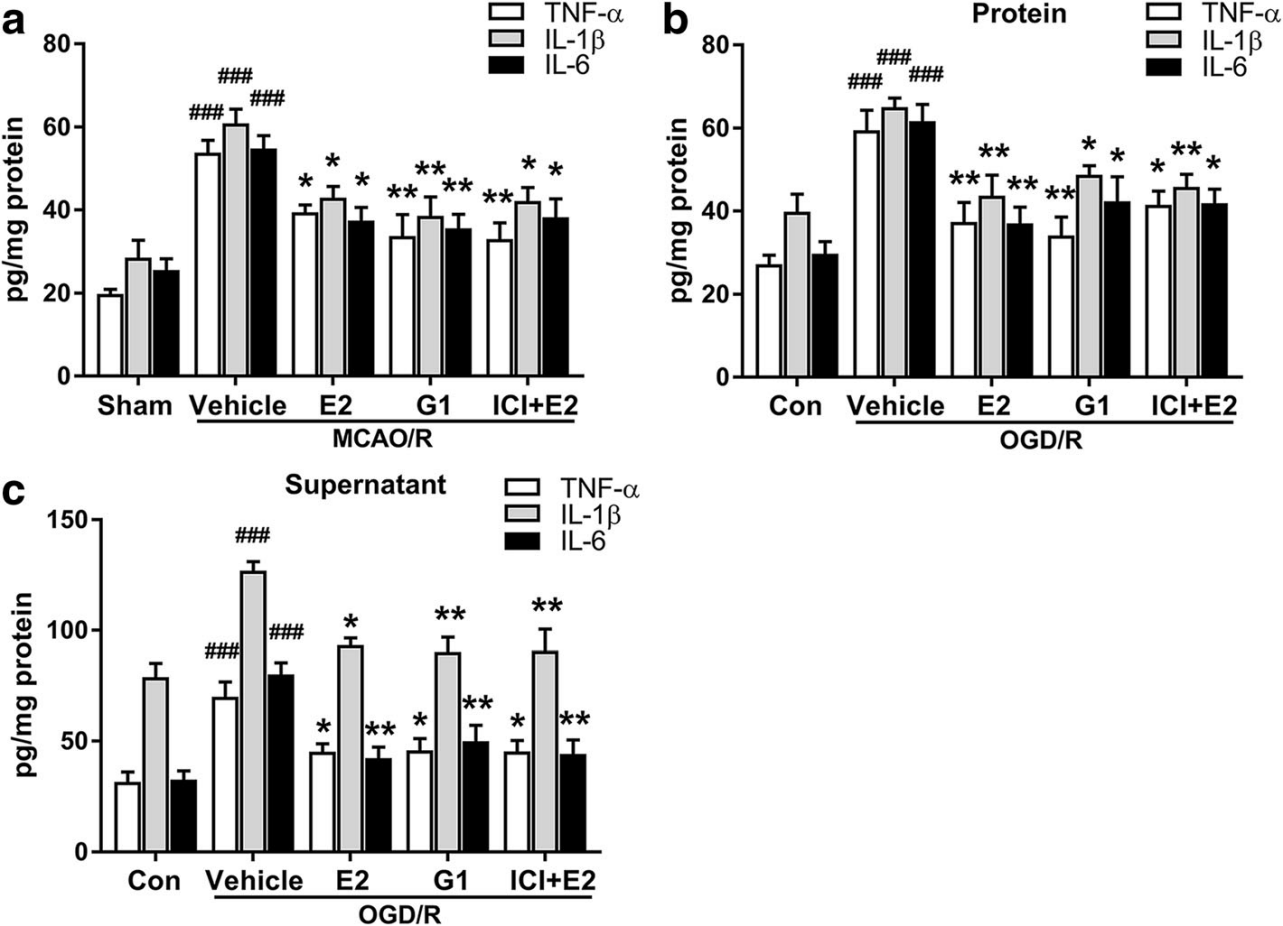
GPR30 activation reduces inflammatory cytokine release after MCAO/R and OGD/R injuries. **a** The levels of the pro-inflammatory cytokines TNF-α, IL-1β, and IL-6 in the ischemic penumbra of OVX mice as detected by ELISA 24 h after reperfusion. The data are expressed as the mean ± SD and analyzed by one-way ANOVA with Tukey’s post-test. ^###^*p* < 0.001 compared with the sham group. *p < 0.05, **p < 0.01 compared with the vehicle group. *n* = 5 per group. **b**, **c** The levels of the pro-inflammatory cytokines TNF-α, IL-1β, and IL-6 in the protein extracts and supernatant of primary microglia after OGD/R injury as detected by ELISA 12 h after the reintroduction of oxygen and glucose. The data are expressed as the mean ± SD and analyzed by one-way ANOVA with Tukey’s post-test. ^###^*p* < 0.001 compared with the con group. *p < 0.05, **p < 0.01 compared with the vehicle group. The data were pooled from five independent experiments. OGD/R oxygen-glucose deprivation/reintroduction

In vivo, the pro-inflammatory cytokines TNF-α, IL-1β, and IL-6 in the penumbra gradually increased and peaked 24 h after ischemia reperfusion (Additional file 1: Figure S3A). In vitro, the 4-h OGD treatment strongly increased the TNF-α, IL-1β, and IL-6 levels in both protein extracts and the supernatant of the microglia (Additional file 1: Figure S3B–C). Thus, the time points of 24 h after ischemia reperfusion and 4-h OGD treatment were selected for subsequent experiments. E2, G1, and ICI + E2 significantly reversed the MCAO/R-induced upregulation of TNF-α, IL-1β, and IL-6 in ischemic penumbra (Fig. 3a). We used primary microglia to further investigate whether GPR30 had anti-inflammatory effects in microglia. E2, G1, and ICI + E2 significantly reversed the OGD/R-induced upregulation of TNF-α, IL-1β, and IL-6 in both protein extracts and the supernatant of microglia (Fig. 3b, c). No significant differences were observed among the E2, G1, and ICI + E2 groups.

### Activating GPR30 in microglia induced neuroprotection against OGD/R injury

**Fig. 4:**
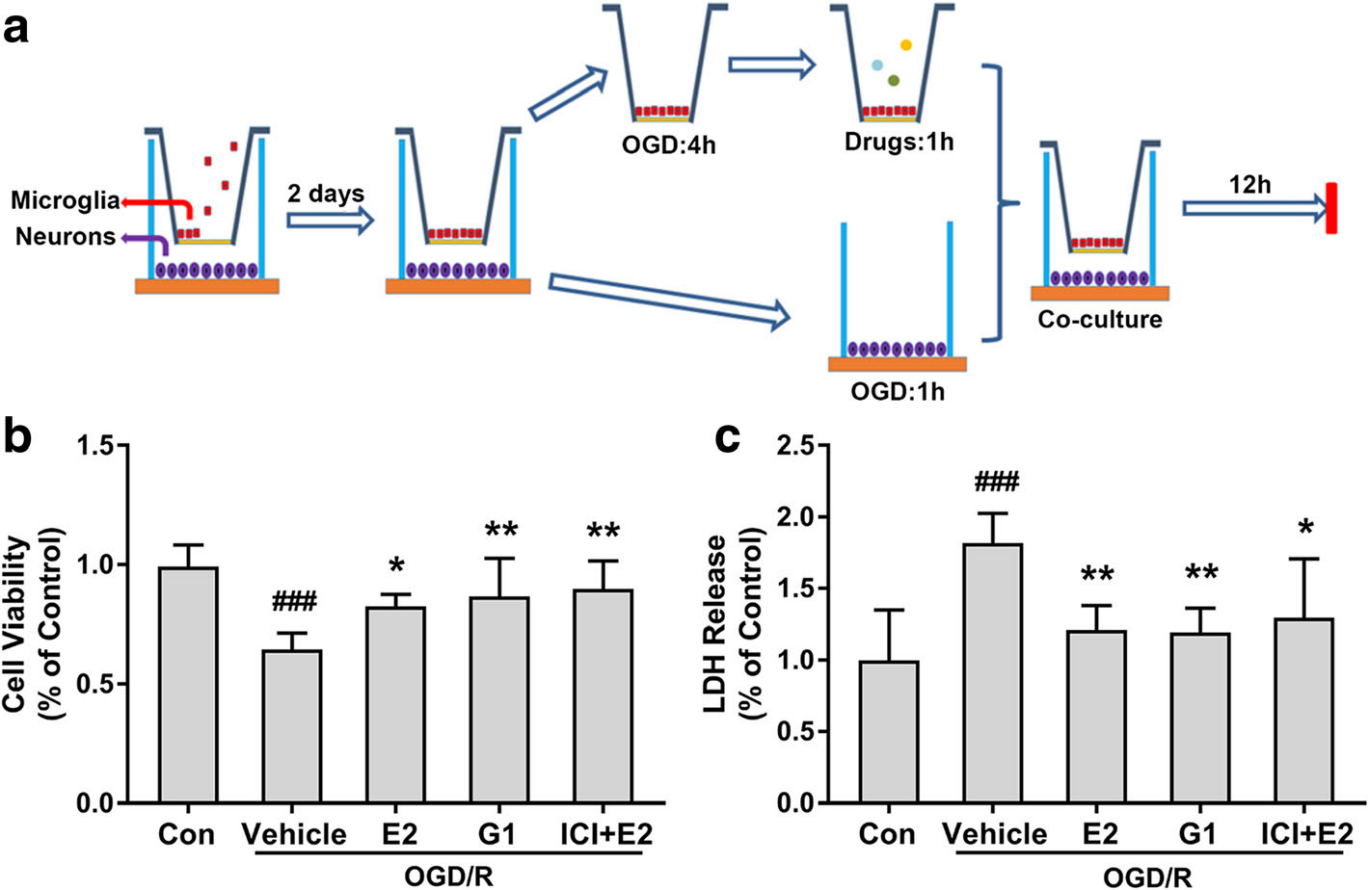
Activating GPR30 in microglia reduces neuronal injury. **a** Neurons and microglia were co-cultured for 2 days and then separated; then, the microglia were subjected to OGD treatment for 4 h and treated with drugs, including E2 (10 nM), G1 (10 nM), and ICI182780 (1 μM), immediately upon reintroduction for 1 h. From the time the microglia received the drug treatments, the neurons were subjected to OGD treatment for 1 h. Then, the neurons and microglia were co-cultured again in new medium for 12 h. CCK-8 cell viability and LDH release assays were performed to detect cell injury. **b** Activation of GPR30 in microglia by E2 and G1 significantly improved the cell viability of neurons. The data are expressed as the mean ± SD and analyzed by one-way ANOVA with Tukey’s post-test. ^###^*p* < 0.001 compared with the con group. *p < 0.05, **p < 0.01 compared with the vehicle group. The data were pooled from six independent experiments. **c** Activation of GPR30 in microglia by E2 and G1 significantly reduced LDH release, indicating that the cell injury was alleviated. The data are expressed as the mean ± SD and analyzed by one-way ANOVA with Tukey’s post-test. ^###^*p* < 0.001 compared with the con group. *p < 0.05, **p < 0.01 compared with the vehicle group. The data were pooled from six independent experiments. LDH lactate dehydrogenase

A neuron-microglia co-culture system was established to investigate the neuroprotection of GPR30 in microglia. During OGD and drug treatments, neurons and microglia were completely separated to avoid activating GPR30 in neurons due to the leakage of drugs through the 0.4-μm pore-sized membrane of the Transwell inserts (see diagram in Fig. 4a). According to the CCK-8 cell viability assay, GPR30 activation in microglia significantly improved the viability of neurons (*p* ˂ 0.0492, vehicle vs E2; *p* = 0.0094, vehicle vs G1; *p* = 0.0028, vehicle vs ICI + E2; Fig. 4b). According to the LDH release assay, the LDH release levels in the E2, G1, and ICI + E2 groups were significantly lower than those in the vehicle group (*p* ˂ 0.0076, vehicle vs E2; *p* = 0.0059, vehicle vs G1; *p* = 0.0259, vehicle vs ICI + E2; Fig. 4c). No significant differences were observed among the E2, G1, and ICI + E2 groups.

### Activating GPR30 relieved microglial activation and inhibited the TLR4/ NF-κB pathway

**Fig. 5:**
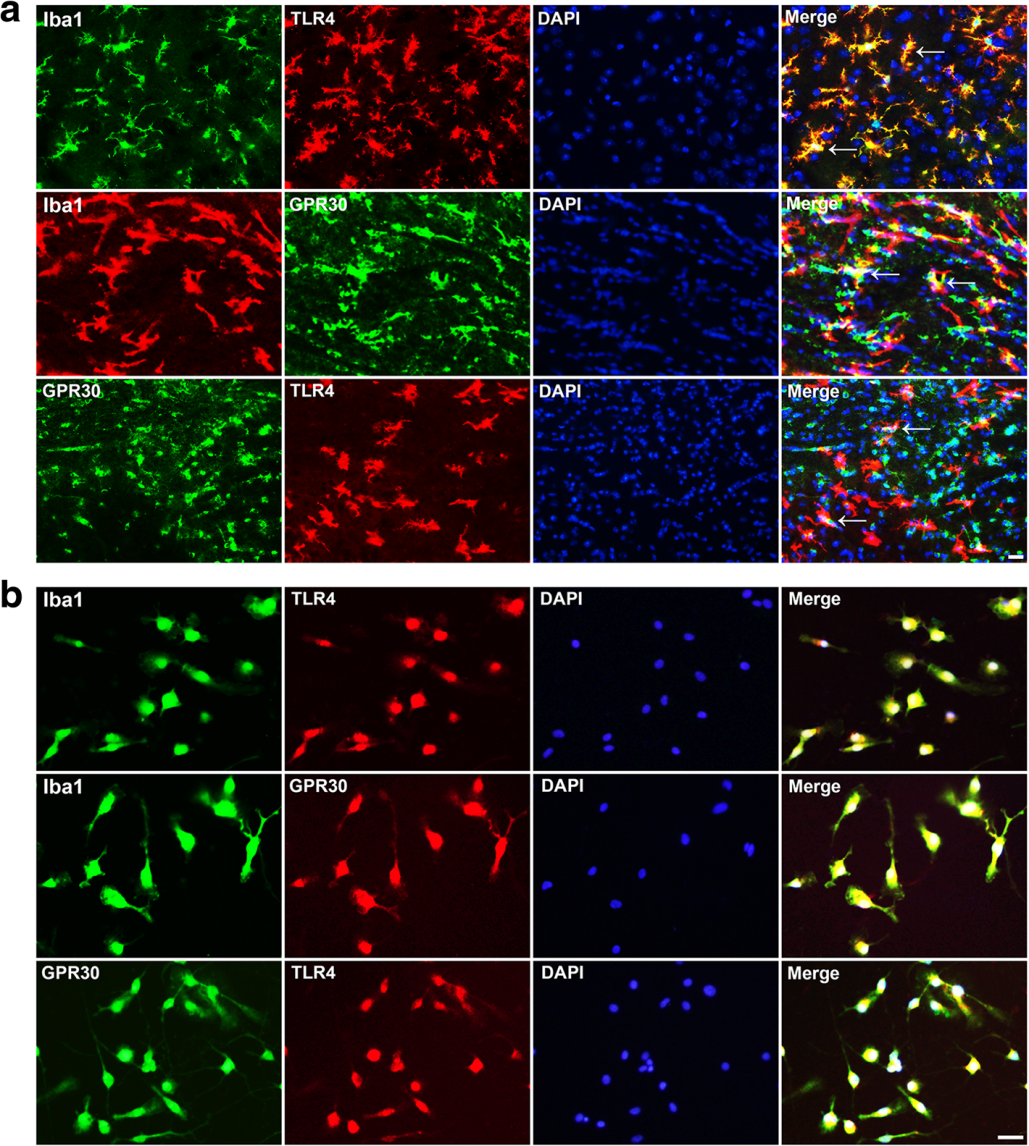
GPR30 co-localized with TLR4 in microglia. **a**, **b** Representative immunofluorescence images showing the co-localization of GPR30 and TLR4 in microglia in the ischemic penumbra of OVX mice and primary mouse microglia. Microglial cells were stained with Iba1. The white arrows show the mutual co-localization of Iba1, TLR4, and GPR30 in microglia. Scale bar = 20 μm

**Fig. 6:**
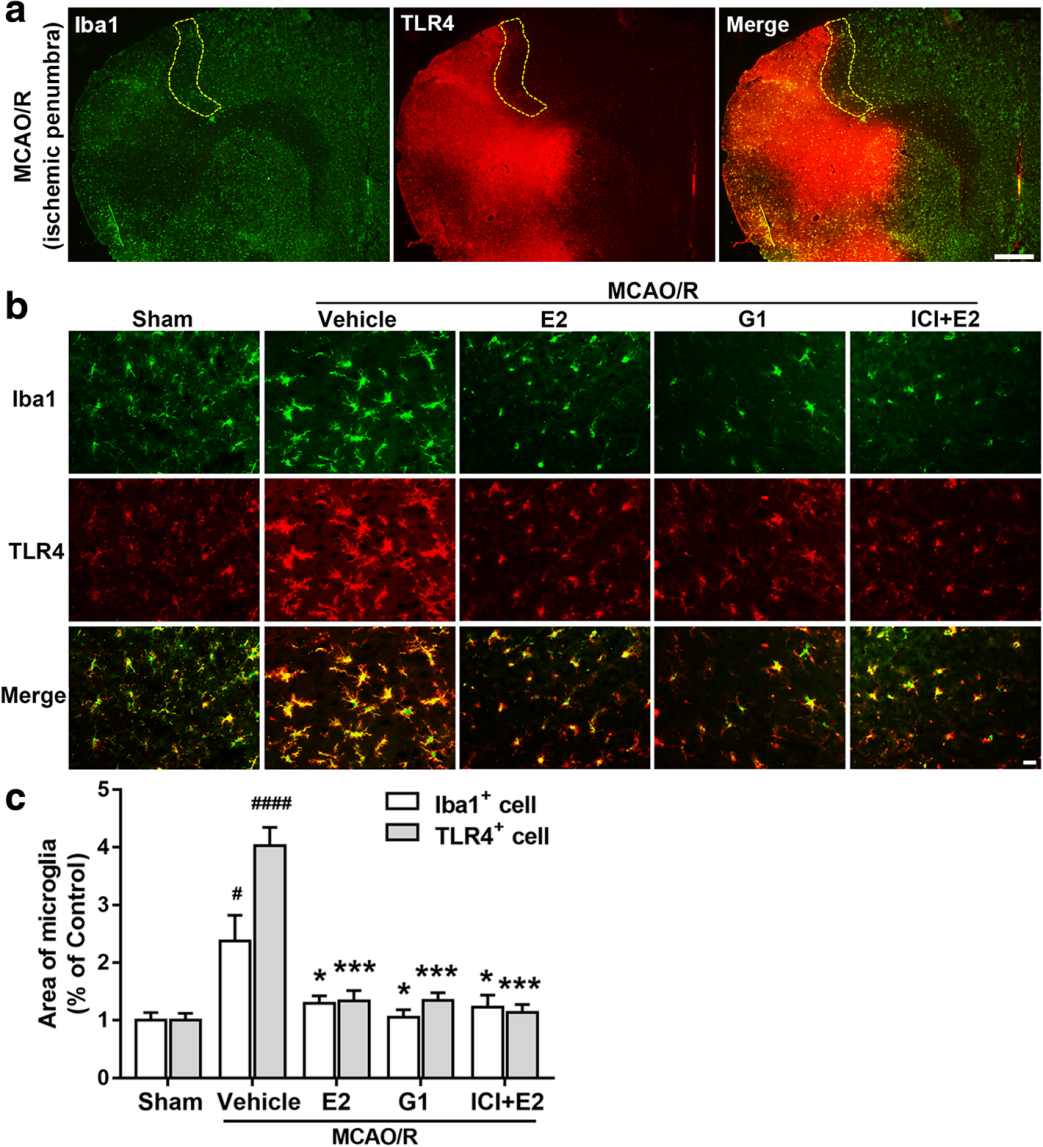
Activating GPR30 relieved microglial activation. **a** Low-magnification micrographs showing microglial cells in ischemic penumbra 24 h after reperfusion. The yellow dotted ring shows the ischemic penumbra area that was addressed. Scale bar = 500 μm. **b** Representative immunofluorescence images showing the morphologies of the microglia cells labeled with Iba1 and TLR4 in microglia 24 h after reperfusion. Scale bar = 20 μm. **c** The average area of single microglial cells labeled by Iba1 and TLR4 in the ischemic penumbra. The data are expressed as the mean ± SD and analyzed by one-way ANOVA with Tukey’s post-test. ^#^*p* < 0.05, ^####^*p* < 0.0001 compared with the sham group. *p < 0.05, ***p < 0.001 compared with the vehicle group. *n* = 6 per group. Five brain sections were analyzed per animal

GPR30 and TLR4 were co-localized in the microglia (Fig. 5). As shown in Fig. 6a, ischemic penumbra was the area between the infarct core and the unaffected tissue. The resting microglia in the sham group had small perinuclear cytoplasms with long, thick processes extending in multiple directions, while the activated microglia in the penumbra of the vehicle group displayed a large soma and short, coarse cytoplasmic processes (Fig. 6b). GPR30 activation by E2 and G1 relieved microglia activation according to visualization of the microglial morphologies (Fig. 6b). Moreover, the average area of single microglial cells labeled by Iba1 was significantly reduced in the E2, G1, and ICI + E2 groups compared with that in the vehicle group (Fig. 6c).

**Fig. 7:**
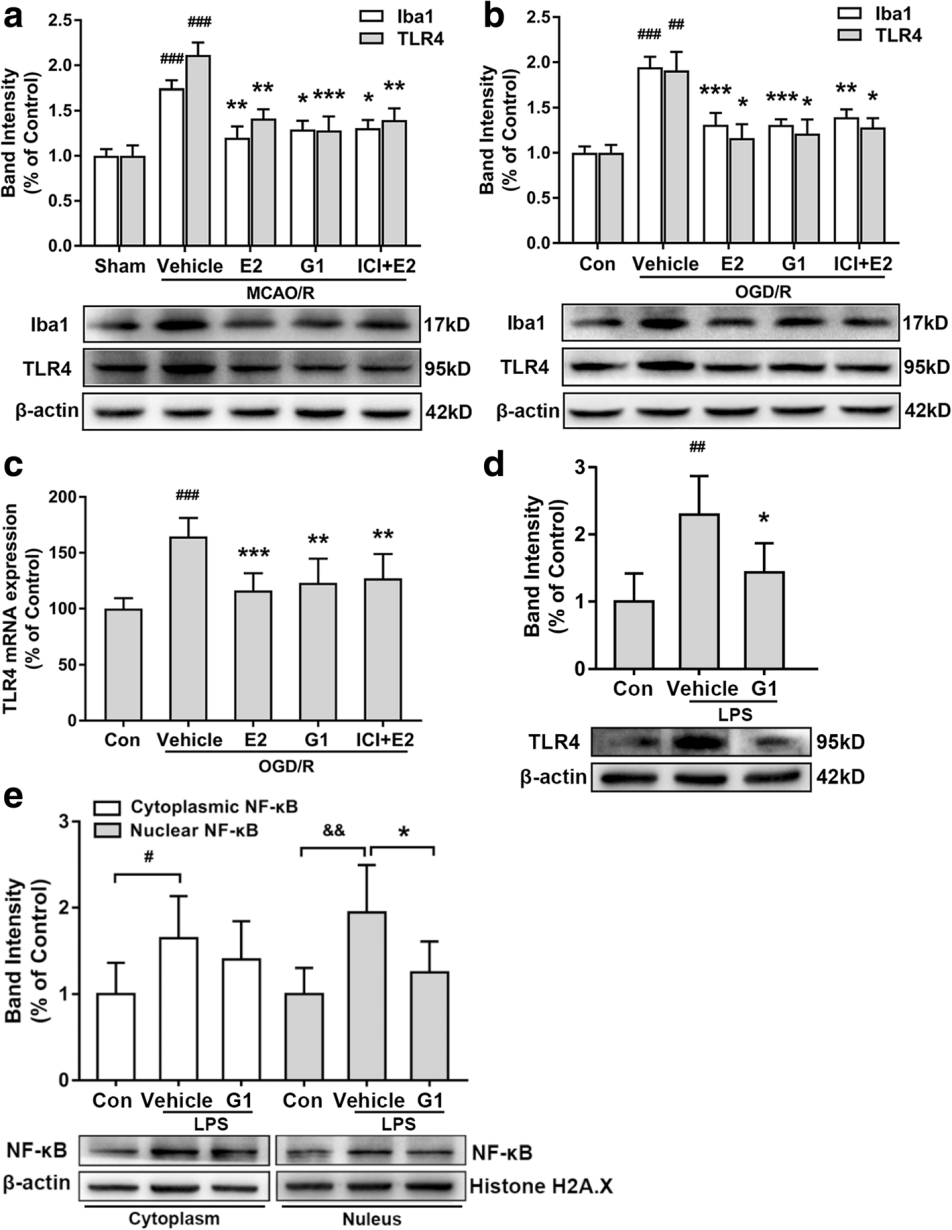
Activating GPR30 inhibited microglial Iba1 protein expression and the TLR4/ NF-κB pathway. **a** Western blotting analysis of Iba1 and TLR4 protein expression in microglia of the ischemic penumbra 24 h after reperfusion. The lower panel shows Iba1 and TLR4 and the corresponding β-actin bands. The histogram in the upper panel shows the results of the densitometric analysis. The data are expressed as the mean ± SD and analyzed by one-way ANOVA with Tukey’s post-test. ^###^*p* < 0.001 compared with the sham group. *p < 0.05, **p < 0.01, ***p < 0.001 compared with the vehicle group. *n* = 6 per group. **b** Western blotting analysis of the protein expression of Iba1 and TLR4 in primary microglia 12 h after the reintroduction of oxygen and glucose. The data are expressed as the mean ± SD and analyzed by one-way ANOVA with Tukey’s post-test. ^##^*p* < 0.01, ^###^*p* < 0.001 compared with the con group. *p < 0.05, **p < 0.01, ***p < 0.001 compared with the vehicle group. The data were pooled from six independent experiments. **c** TLR4 mRNA expression levels in primary microglia as detected by real-time PCR 12 h after the reintroduction of oxygen and glucose. The data are expressed as the mean ± SD and analyzed by one-way ANOVA with Tukey’s post-test. ^###^*p* < 0.001 compared with the con group. **p < 0.01, ***p < 0.001 compared with the vehicle group. The data were pooled from six independent experiments. **d** Western blotting analysis of TLR4 protein expression in LPS-activated primary microglia 12 h after G1 treatment. The data are expressed as the mean ± SD and analyzed by one-way ANOVA with Tukey’s post-test. ^##^*p* < 0.01 compared with the con group. ^*^p < 0.05 compared with the vehicle group. The data were pooled from five independent experiments. **e** Cytoplasmic and nuclear levels of NF-kB in LPS-activated primary microglia 12 h after G1 treatment as detected by Western blot. The data are expressed as the mean ± SD and analyzed by one-way ANOVA with Tukey’s post-test. ^#^*p* < 0.05, ^&&^*p* < 0.01 compared with the indicated con group. *p < 0.05 compared with the vehicle group. The data were pooled from five independent experiments. LPS lipopolysaccharide

The 24-h time point after ischemia reperfusion and the 4-h OGD treatment were selected to investigate the influence of GPR30 on the expression of Iba1 (Additional file 1: Figure S4). As shown in Fig. 7a, the activation of GPR30 significantly reduced the expression of Iba1 in ischemic penumbra (*p* = 0.0037, vehicle vs E2; *p* = 0.0191, vehicle vs G1; *p* = 0.0242, vehicle vs ICI + E2). Primary microglia were used to further confirm that the activation of microglial GPR30 significantly reduced Iba1 expression levels (*p* = 0.0009, vehicle vs E2; *p* = 0.0008, vehicle vs G1; *p* = 0.0039, vehicle vs ICI + E2; Fig. 7b). No significant differences were observed among the E2, G1, and ICI + E2 groups.

We detected TLR4 expression levels to further investigate whether the anti-inflammatory effect of GPR30 was mediated by TLR4. The 24-h time point after ischemic reperfusion and the 4-h OGD treatment were selected for subsequent experiments (Additional file 1: Figure S4). The TLR4 protein levels were significantly reduced in microglia of the ischemic penumbra (*p* = 0.0009, vehicle vs G1; *p* = 0.0045, vehicle vs ICI + E2; Fig. 7a) and primary microglia (*p* = 0.0118, vehicle vs E2; *p* = 0.0207, vehicle vs G1; *p* = 0.0443, vehicle vs ICI + E2; Fig. 7b) after the E2, G1, and ICI182780 + E2 treatments. The real-time PCR results showed that E2 and G1 inhibited the expression of TLR4 mRNA in microglia (*p* = 0.0006, vehicle vs E2; *p* = 0.0033, vehicle vs G1; *p* = 0.0087, vehicle vs ICI + E2; Fig. 7c). To further confirm the downregulation of TLR4 via GPR30 activation, we detected NF-κB activity in LPS-activated microglia 12 h after G1 treatment. As shown in Fig. 7d, the TLR4 protein levels were significantly increased in microglia after 12 h of exposure to LPS and significantly reduced in LPS-activated microglia after the activation of GPR30 (*p* = 0.0037, vehicle vs con; *p* = 0.0289, G1 vs vehicle). Interestingly, NF-κB expression was significantly increased in both the cytoplasm and the nucleus, which demonstrated the nuclear translocation of NF-κB after LPS exposure (*p* = 0.0436 and *p* = 0.0096, vehicle vs the corresponding con; Fig. 7e). G1 treatment significantly reduced NF-κB expression in the nucleus without influencing its expression in the cytoplasm (*p* = 0.0448, G1 vs vehicle; Fig. 7e), which showed that GPR30 had an inhibitory effect on the TLR4/NF-κB pathway.

## Discussion

We investigated the roles of microglial GPR30 in the neuroprotection of estrogen against ischemic injury in vitro and in vivo. We found that GPR30 was highly expressed in microglia and significantly increased after ischemic injury. Meanwhile, the activation of GPR30 significantly improved the neurological deficit and alleviated neuronal injuries. Moreover, the activation of GPR30 significantly reduced the TLR4 expression levels and NF-κB activity, relieved microglial activation, and decreased the release of TNF-α, IL-1β, and IL-6 in ischemic penumbra and microglia. Thus, microglial GPR30 exerts neuroprotective effects against cerebral ischemic injury by inhibiting TLR4-mediated microglial inflammation, suggesting that GPR30 may be a potential therapeutic target in the treatment of ischemic stroke.

GPR30 was expressed in microglia, which was confirmed by RNA-sequencing transcriptome studies using primary microglia or microglial cell lines and immunofluorescence staining performed in our study [18, 19]. GPR30 participates in the acute neuroprotection of estrogen and regulates neuronal functions, such as neurotransmitter release and hippocampal synaptic plasticity [20]. An acute application of the GPR30 agonist G1 following ischemic injury reduced neuronal damage in both aged rats and adult mice [7, 9]. GPR30 activation also improved neuronal survival in the hippocampus and striatum following global cerebral ischemia [8]. The acute neuroprotective effects of GPR30 have great clinical significance because estrogenic compounds could be used to specifically activate GPR30 without the undesirable side effects of estrogen.

Estrogen provided neuroprotection by inhibiting inducible nitric oxide (iNOS) and TNF-α in microglia [21, 22], suggesting that estrogen participates in the brain’s immune system [23, 24]. However, the receptor mediating the anti-inflammation effect of estrogen remains controversial. Because both ERα and ERβ are expressed in immunocytes, several studies have indicated that ERα plays a key role in inhibiting NF-κB and iNOS expression in macrophages [23, 25], while other studies have demonstrated that ERβ plays an anti-inflammatory role in microglia, macrophages, and astrocytes [26]. Interestingly, several studies also indicated that E2 downregulated TNF-α via the GPR30-mediated Ca^2+^ signaling pathway in macrophages [27]. We found that the activation of microglial GPR30 reduced the release of TNF-α, IL-1β, and IL-6 and decreased microglial activation, further confirming that GPR30 also mediates the anti-inflammatory effect of estrogen. However, whether microglial GPR30 has a higher affinity for ERα or ERβ requires further investigation.

As innate immunocytes, microglia were excessively activated and released robust inflammatory cytokines, such as IL-1, IL-6, TNF, and iNOS, immediately after ischemic stroke, which contributed to neuronal apoptosis and thus aggravated brain injury [28]. Studies have shown that TNF-α, IL-1β, and IL-6 expression is elevated 0.5, 6, and 3.5 h after stroke, respectively [29]. The fact that inflammatory cytokines were produced by microglia centrally places these cells in potential future stroke therapy [28]. In our study, we found that activating microglial GPR30 relieved inflammatory responses and alleviated neuronal injury. However, neuronal GPR30 activation may have indirect effects on microglia activation because GPR30 is also expressed in neurons and modulates synaptic plasticity in the mouse hippocampus [30]. Therefore, a mouse model with a conditional knockout of the gpr30 gene in microglia is needed to better verify the specific neuroprotective effect of microglial GPR30 in the future. Moreover, whether the neuroprotection effect of microglial GPR30 has priority over neuronal GPR30 and the interrelation between the activation of neuronal GPR30 and microglial GPR30 requires further investigation.

GPR30 performed its functions by targeting downstream molecules. The PI3K/Akt pathway is involved in the anti-apoptotic effect of GPR30 in spinal neurons [31]. G1 regulates hippocampal synaptic plasticity by inducing brain-derived neurotrophic factor (BDNF) release [20]. Moreover, GPR30 ameliorated blood-brain barrier permeability by inhibiting vascular endothelial growth factor A expression [32]. In our study, microglial GPR30 exerted neuroprotective effects by inhibiting TLR4-mediated microglial inflammation. Several potential pathways may be involved in GPR30-induced TLR4 downregulation in microglia. First, GPR30 may indirectly modulate the transcriptional activity of TLR4 by activating the second messenger signaling cascades. GPR30 activation has been shown to lead to the activation of adenylate cyclase and a subsequent increase in cAMP levels [33]. GPR30 activation also induced the activation of PI3-kinase/Akt-dependent signaling and transactivation of the epidermal growth factor receptor via Src protein kinase [33–35]. Moreover, GPR30 increased Bcl-2 and BDNF by regulating the PI3K/Akt and MAPK/ERK signaling pathways [36]. Whether these second messenger signaling molecules are required for the GPR30-mediated downregulation of TLR4 in microglia needs to be determined. Second, GPR30 may inhibit TLR4 expression via “bridge molecules,” i.e., Rab proteins. Members of the Rab protein family are small GTPases that are widely expressed in the CNS [37]. Rab8b is a GPR30-binding protein that has been confirmed by a mammalian two-hybrid system [19]. More importantly, Rab proteins participate in TLR4-mediated inflammation. Rab8a and Rab10 decreased membrane TLR4 expression and its downstream pro-inflammatory cytokines [38, 39], and Rab7b reduced TLR4 expression levels in macrophages [37]. These studies indicate that Rab proteins may act as a molecular link between GPR30 and TLR4.

Our study does have some limitations. First, using a mouse model with a conditional knockout of the gpr30 gene in microglia could better verify the effect of microglial GPR30. Second, the mechanisms underlying the GPR30-mediated downregulation of TLR4 expression require further clarification.

## Conclusions

Our study indicates that microglial GPR30 plays a key role in the TLR4-mediated inflammatory response and in the acute neuroprotective effects of estrogen after ischemic stroke, which enhances our comprehension of GPR30’s function in the CNS. Most importantly, our study suggests that GPR30 may be a potential therapeutic target for the treatment of acute ischemic stroke.

## Additional file

Additional file 1: **Table S1.** Arterial gas analysis in sham and MCAO groups. Figure S1 The identification of primary microglial cells in culture. Figure S2 The expression of GPR30 protein increases after ischemic injury. Figure S3 The levels of TNF-α, IL-1β, and IL-6 increase after ischemia/hypoxic injury. Figure S4 The protein expression of Iba1 and TLR4 increases after ischemia/hypoxic injury. (DOCX 1090 kb)

## Abbreviations

BDNF: Brain-derived neurotrophic factor
CCK8: Cell counting kit-8
CNS: Central nervous system
DMEM: Dulbecco’s Modified Eagle’s Medium
DMSO: Dimethyl sulfoxide
E2: 17-β-estradiol
ERα: Estrogenic receptors α
ERβ: Estrogenic receptors β
GPR30: G protein-coupled receptor 30
iNOS: Inducible nitric oxide
LDH: Lactate dehydrogenase
LPS: Lipopolysaccharide
MCAO: Middle cerebral artery occlusion
OGD: Oxygen-glucose deprivation
OVX: Ovariectomized
TLR4: Toll-like receptor 4
TTC: 2,3,5-Triphenyl-2H-tetrazoliuM chloride
TUNEL: Terminal deoxynucleotidyl transferase-mediated dUTP nick-end labeling
