# Supplementary material for: The novel estrogenic receptor GPR30 alleviates ischemic injury by inhibiting TLR4-mediated microglial inflammation
Source: J Neuroinflammation. 2018 Jul 12;15:206. doi: 10.1186/s12974-018-1246-x (PMC6043971; doi:10.1186/s12974-018-1246-x)
Supplement: Supplementary file 1 — Table S1. Arterial gas analysis in sham and MCAO groups. Figure S1 The identification of primary microglial cells in culture. Figure S2 The expression of GPR30 protein increases after ischemic injury. Figure S3 The levels of TNF-α, IL-1β, and IL-6 increase after ischemia/hypoxic injury. Figure S4 The protein expression of Iba1 and TLR4 increases after ischemia/hypoxic injury. (DOCX 1090 kb) [file 12974_2018_1246_MOESM1_ESM.docx]

**Supplemental Methods and Figures**

***Measurement of physiological parameters***

The blood gas was analyzed by collecting blood samples from the carotid artery of separate mice at 10 min before MCAO, during MCAO and 10 min after reperfusion and was confirmed using an OMNI Modular System (Rapidlab 1260, Bayer HealthCare, Uxbridge, UK).

**Table S1 Arterial gas analysis in Sham and MCAO groups**

| Group | T (℃) | PH | PaO_2_ (mmHg) | PaCO_2_ (mmHg) |
| --- | --- | --- | --- | --- |
| 10 min before MCAO: |  |  |  |  |
| Sham | 37.2±0.1 | 7.34±0.05 | 120.47±9.24 | 36.68±5.69 |
| MCAO | 37.6±0.1 | 7.36±0.04 | 116.29±11.79 | 37.61±4.85 |
| During MCAO: |  |  |  |  |
| Sham | 36.9±0.3 | 7.35±0.02 | 117.83±8.52 | 37.88±5.81 |
| MCAO | 37.3±0.2 | 7.34±0.06 | 119.16±10.68 | 38.19±5.47 |
| 10 min after reperfusion: |  |  |  |  |
| Sham | 37.3±0.2 | 7.37±0.03 | 121.75±9.21 | 39.23±4.62 |
| MCAO | 37.1±0.2 | 7.36±0.05 | 120.61±9.39 | 38.84±4.13 |

Data of blood gas analysis showing the PH, PaO_2_, PaCO_2_ as well as the body temperature of the separate mice 10 min before, during MCAO operation and 10 min after reperfusion. All values were expressed as mean±SD, n=5. There were no significant differences between the Sham group and the MCAO group.


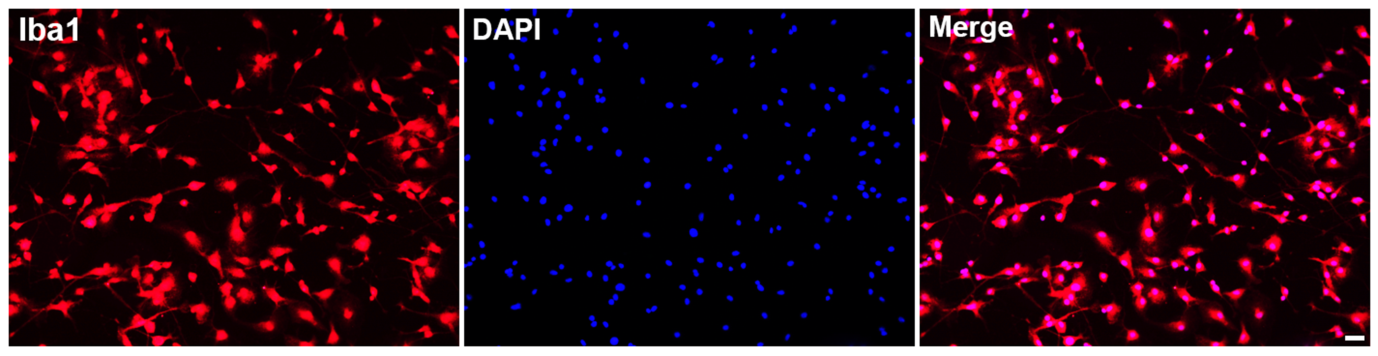


**Figure S1 The identification of primary microglial cells in culture**

The purity of the microglial cells in culture were confirmed by staining with the microglia marker Iba1 and DAPI. Scale bar=20 μm.


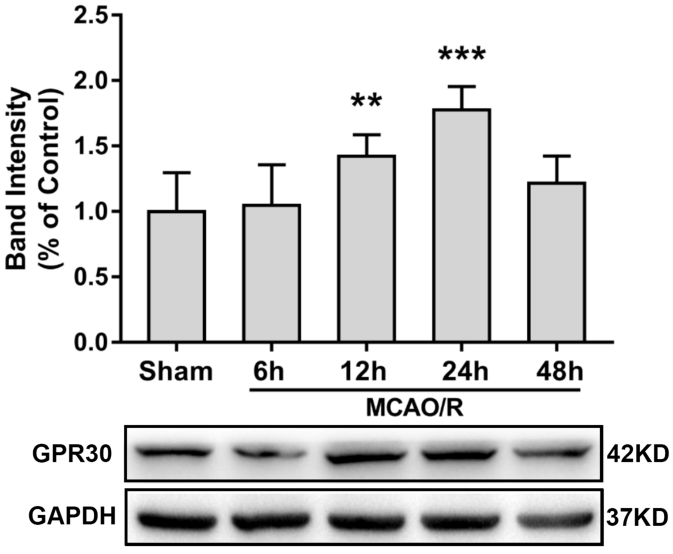


**Figure S2 The expression of GPR30 protein increases after ischemic injury**

Western blotting analysis of the expression levels of GPR30 protein in ischemic penumbra of the OVX mice 6 hours, 12 hours, 24 hours and 48 hours after reperfusion. The lower panel shows GPR30 and the corresponding GAPDH bands. The upper panel histogram shows the results of the densitometric analysis. The data were expressed as the mean±SD and analyzed by one-way ANOVA with Dunnett’s post-test. ***p*<0.01, ****p*<0.001 compared with the Sham group. n=6 per group.


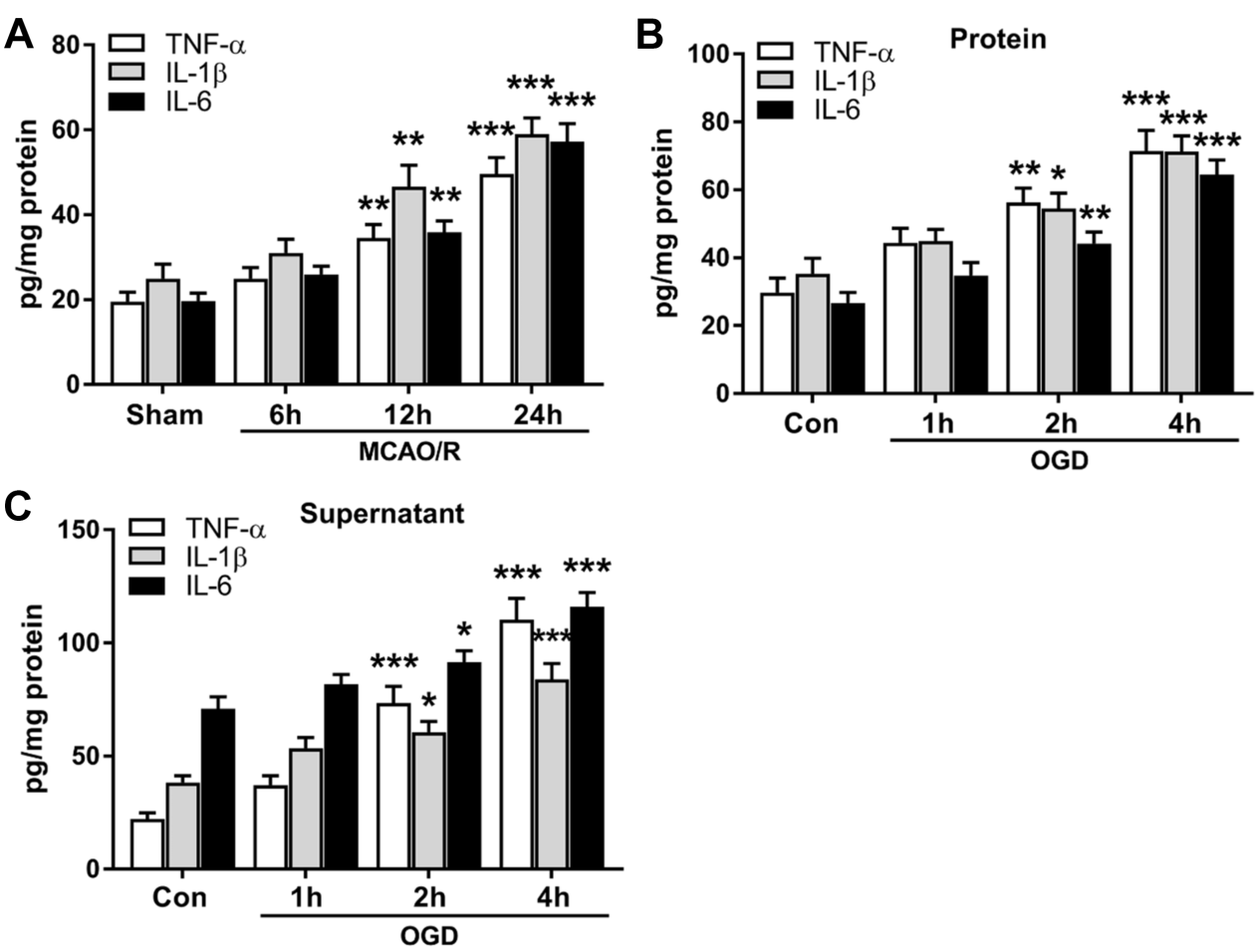


**Figure S3 The levels of TNF-α, IL-1β and IL-6 increase after ischemia/hypoxic injury**

(A) The levels of the pro-inflammatory cytokines TNF-α, IL-1β and IL-6 in ischemic penumbra of the OVX mice were detected by ELISA 6 hours, 12 hours and 24 hours after reperfusion. The data were expressed as the mean±SD and analyzed by one-way ANOVA with Dunnett’s post-test. ***p*<0.01, ****p*<0.001 compared with the Sham group. n=5 per group. (B-C) The levels of the pro-inflammatory cytokines TNF-α, IL-1β and IL-6 in the protein extracts and supernatant of primary microglia subjected to OGD treatments for 1 hour, 2 hours and 4 hours and were detected by ELISA 12 hours after reintroduction of oxygen and glucose. The data were expressed as the mean±SD and analyzed by one-way ANOVA with Dunnett’s post-test. **p*<0.05, ***p*<0.01, ****p*<0.001 compared with the Con group. The data were pooled from five independent experiments.


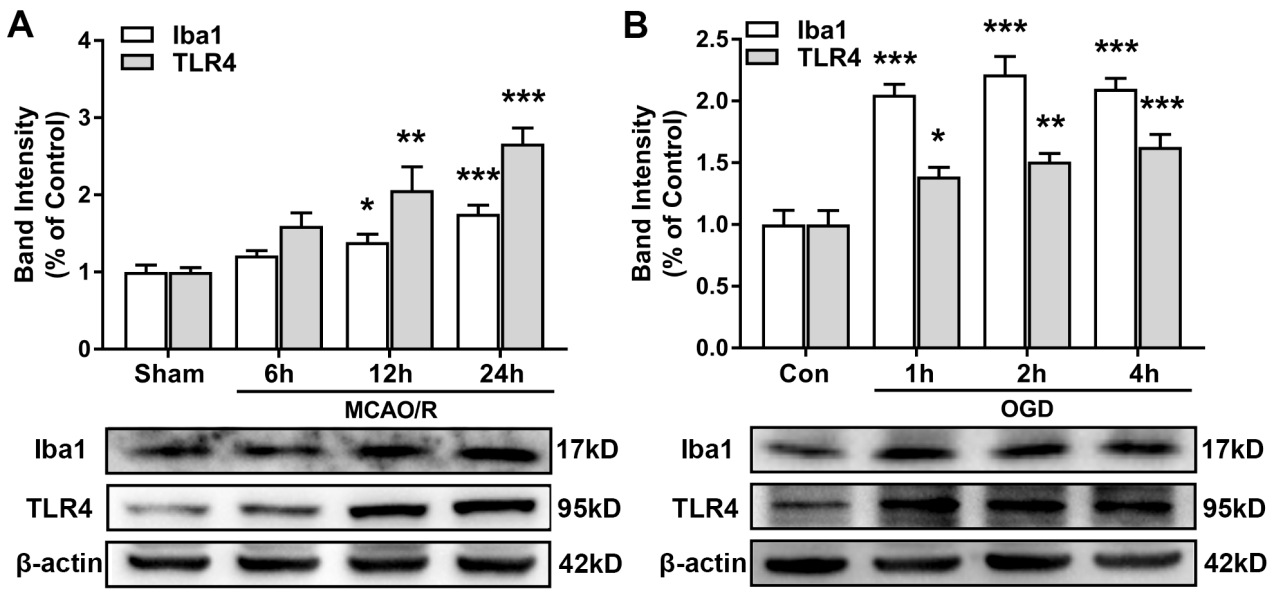


**Figure S4 The protein expression of Iba1 and TLR4 increases after ischemia/hypoxic injury**

(A) Western blotting analysis of the expression levels of Iba1 and TLR4 proteins in ischemic penumbra of the OVX mice 6 hours, 12 hours and 24 hours after reperfusion. The lower panel shows Iba1 and TLR4 and the corresponding β-actin bands. The upper panel histogram shows the results of the densitometric analysis. The data were expressed as the mean±SD and analyzed by one-way ANOVA with Dunnett’s post-test. **p*<0.05, ***p*<0.01, ****p*<0.001 compared with the Sham group. n=6 per group. (B) Western blotting analysis of the expression levels of Iba1 and TLR4 proteins in microglia subjected to ODG treatments for 1 hour, 2 hours and 4 hours and assessed at 12 hours after reintroduction of oxygen and glucose. The lower panel shows Iba1 and TLR4 and the corresponding β-actin bands. The upper panel histogram shows the results of the densitometric analysis. The data were expressed as the mean±SD and analyzed by one-way ANOVA with Dunnett’s post-test. **p*<0.05, ***p*<0.01, ****p*<0.001 compared with the Con group. The data were pooled from six independent experiments.
